# Periscope

**Published:** 1887-12

**Authors:** 


### PERISCOPE.

### MEDICINE.

**Tuberculosis of the Bulb in a Child.**—Mons. Bonnerville Isch-Wall reports an interesting case of this somewhat rare lesion. The patient came of a family with bad nervous history, but non-phthisical. The first appearance of illness came on when three years old, with digestive troubles, night-terrors, and change of character. Eight months after, the respiration became sighing and hurried, there were attacks of giddiness and a transient strabismus of the left eye, with weakness of the legs and some fever. The child appeared to become stupid. After a remission of six weeks the above symptoms reappeared, there now being marked dyspnœa and left internal strabismus. On admission, the child could with difficulty walk and speak. Shortly afterwards all power of speech and of movement of the limbs was lost: there was dysphagia, the expression became anxious and suffering, and a plaintive cry was frequently uttered. The pulse was very irregular, and the temperature slightly raised. These symptoms gradually became worse, but with notable remissions occurring almost daily. Paraplegia present one day disappeared the next, and an area of skin before hypersensitive became anæsthetic. For a few days preceding the fatal termination there was notable emaciation, retraction of the abdomen, and diminution of the urinary secretion. Dysphagia, constipation, stiffness of the cervical spine, paralysis and contracture of the limbs became more marked; and for two days before death the temperature steadily rose until it attained the height of 108° F., when death occurred.

The autopsy showed old caseous nodules, some partly cretaceous, in the apices of both lungs. The bulb was enlarged, and contained also a number of caseating tubercular nodules from the size of a pea to that of a nut. There was double descending sclerosis of the crossed pyramidal tracts in the spinal cord. The rest of the brain and the other organs were normal.

The authors give a useful collection of ten recorded cases of a similar kind, and point out how extremely the symptoms of bulbar tumours vary in them. The most constant symptom is strabismus; vomiting comes next in order of frequency, but may be absent; and crossed paralysis, though very frequent, may not occur, as in the above quoted case. They would class the cases in three groups: (1) Tuberculous lesions of the bulb, giving rise to no symptoms until just before death, which may be caused by a concurrent affection of some other organ; (2) the disease advancing slowly, with intermittent aggravations from time to time; and (3) following a steadily progressive course.

The high temperature observed towards the end in this case was not noted in any of the other instances quoted, in several of which it remained subnormal throughout the duration of the illness.—*Le Progrès médical*, August 20th, 1887.

**Treatment of Cirrhosis.**—M. Lancereaux was led to adopt the treatment of alcoholic cirrhosis by iodide of potassium from observations made as to its good effects in cases of syphilitic and malarial disease of the liver. He argued that since, histologically, all these diseases are characterised by the overgrowth of cellular elements of the same kind, if iodide of potassium relieved the two latter, it should also be beneficial in cases of the first kind. The first case of alcoholic cirrhosis was that of a young man who had been a heavy drinker, and who was apparently dying of advanced cirrhosis, characterised by diminution in size of the liver, ascites, œdema of the legs, emaciation, atrophy of muscles, bilious vomiting, &c. He was put upon a milk diet, with iodide of potassium, and frictions to the skin. To M. Lancereaux's surprise, improvement in the general condition was soon manifest, though the ascites continued to augment until paracentesis became necessary. The ascites returned to nearly its former extent after paracentesis, then completely disappeared; and three months from the commencement of treatment, there was no fluid in the abdomen, and the patient had regained his flesh and strength, and considered himself well. Five years afterwards, having reformed his mode of life, he still remained well. He gives details of three other cases cured by the same means, and is confident that by these measures atrophic cirrhosis is not only capable of amelioration, but also of cure. In the so-called hypertrophic form of cirrhosis the results are not so good, and this he attributes to the facts that the glandular cells in this form are frequently largely destroyed, and also undergo extensive fatty degeneration. In alcoholic cirrhosis attended with persistent jaundice no benefit was obtained; but with these exceptions treatment failed in only one case, and here the *post-mortem* examination showed that an error of diagnosis had been made, and that the case was one of peri-hepatitis.

The rapidity of the cure and the duration of treatment necessarily vary with the more or less advanced stage of the malady. In favourable cases, improvement generally begins to be manifest in about fifteen days, with increase of the urinary secretion and diminution of œdema and ascites. At the same time dilatation of the subcutaneous abdominal veins and enlargement of the spleen gradually disappear, while the patient gains flesh and strength, and digestive disturbances improve. These results obtain in from six weeks to three months; but on account of the tendency of the disease to re-establish itself, the treatment should be continued longer, from some months to one year in bad cases, if they wish to avoid a recurrence. At the same time the patient must abstain from all forms of alcohol, and observe the strictest dietary and hygienic regulations, and the danger of departing from these precautions must be impressed upon them. M. Lancereaux gives the case of a man who was relieved of all symptoms, and remained well for a year, but then relapsed into his former drunken habits, and died of a return of the disease. He attributes the good effects of the iodide to its power of preventing the growth and development of the young connective tissue, and finally insists on the importance of a raw-milk diet during the time of treatment.—*Gazette des hôpitaux*, September 1st, 1887. J. Michell Clarke, M.B.

### SURGERY.

**Endoscopy.**—Dr. L. L. Friedrich read a paper on this subject before the Medical Society of the District of Columbia. After referring to the slight amount of use made of the endoscope, he describes the instrument as no better than a straight tube, blackened on the inside and furnished with an obturator. It is made either of metal or hard rubber; the latter is to be preferred when caustics are used. To make a thorough examination the instrument should be well warmed and oiled, and introduced gently into the membranous urethra. The obturator is then withdrawn, the oil and mucus wiped away from the presenting membrane at the bottom of the tube by means of a little pledget of cotton at the end of a probe. The illumination is then brought to bear directly on each portion of the membrane as the tube is withdrawn. The ordinary laryngoscope mirror answers every purpose. A little practice is required to distinguish healthy from diseased tissue. As with the ophthalmoscope, all at first appears red.

The healthy mucous membrane has a pale pink colour, and contrasts strongly with congested spots, which are of a vinous red without polish: granulations also are readily recognised by the practised eye. By means of the endoscope strong solutions can be applied directly, and with accuracy, to the diseased spots.

The urethral chancre is not very uncommon, and can be detected by the endoscope. Professor Von Dittel showed that by means of the endoscope and electric light the interior of the bladder could be thoroughly explored. Professor Janowisky says that the endoscope is sometimes valuable in prognosis, and as an aid to diagnosis, especially in strictures of a wide calibre, as described by Otis.—*The Journal of the American Medical Association*, September 3rd, 1887.

**Surgical Scarlatina.**—Dr. Hoffa, of Würzburg, in a lecture recently published in Volkmann's *Sammlung Klinischer Vorträge*, points out that in many of the numerous recorded cases of so-called surgical scarlatina there is no evidence of scarlatinal infection. These cases, it is stated, may be arranged under four classes. In the first the rash is nothing more than erythema, due to vaso-motor disturbance, which appears early and vanishes rapidly.

The second class is made up of cases of toxic erythema, occurring in the course of twenty-four or forty-eight hours after some cutting operation, and after injury with or without wounding of the skin. This rash resembles any erythematous medicinal rash, and is accompanied by gastric disturbance and high fever. It occurs in the form of fine red points, or large isolated patches with intervening clear spaces of healthy skin. As a rule it disappears in twenty-four hours, and is not followed by desquamation. It is probably caused by the absorption of wound secretions, or the pressure of crushed tissue in the circulation, or may be due in some cases to the action of fibrin ferment. The rash in the third group is the result of septicæmic or pyæmic infection, and is of much more serious import than the preceding groups. It usually occurs in the form of a disseminated erythema, often associated with miliaria and pustules, with purpuric spots, and with ecthyma. There is one form of pyæmic rash which it is very difficult to distinguish from true scarlatina. On the third, fifth, or seventh day after the first attack of rigors, this rash usually appears at the wound or open surface, and in the course of the two following days spreads as a bright and evenly-diffused redness, often limited to one side of the trunk. This rash disappears for a moment under digital pressure, and is usually accompanied by œdematous swelling of the skin. It vanishes in the course of the fourth or fifth day, and is often followed by desquamation, and sometimes by purulent infiltration of the subcutaneous cellular tissue. This variety of exanthem is probably due to capillary embolism through micro-organisms.

Besides these three groups, there is a fourth, which comprises cases in which the rash is really the result of the infection of the organism with the poison of scarlatina. Dr. Hoffa says that the diagnosis of scarlatina can only be accepted when, in addition to the characteristic exanthem, there is present at least one of the other morbid conditions that go to form the group of symptoms of the eruptive fever; viz., angina, swelling of the submaxillary glands, desquamation or nephritis. The diagnosis will be rendered certain if the patient has been exposed to the infection of scarlatina, or if he has transmitted the disease to others. These cases may be subdivided into two classes: first, those patients who with a wound take the infection in the ordinary manner, through the respiratory or digestive tract; and secondly, those cases in which the infection enters by the wound and spreads from it. The author believes that the tendency of the subject of an open wound to contract scarlatina is due to the circumstance, that the wound is a convenient place for the entrance and development of the micro-organisms.

Sir James Paget believes that in these cases the period of incubation is shortened. The name surgical scarlatina should only be applied to those cases in which the scarlatinal infection starts from the wound. In these cases the characteristic symptoms of scarlatina are often much modified, but probably not more so than in ordinary cases of scarlatina without a wound, which often deviates in many ways from the typical course of the disease.—*The Medical Record*, October 8th, 1887.

**Treatment of Anal Fissures and Hæmorrhoids by Gradual Dilatation.**—Dr. H. O. Walker has treated upwards of fifty cases of anal fissures and hæmorrhoids by this method. He states that anal fissure ranks third in frequency among diseases of the rectum, and is due principally to the passage of hardened fæces through the sphincters. Monod, Verneuil, Kelsey, and Allingham have all recommended forcible dilatation for hæmorrhoids, either simply or combined with other measures. Forcible dilatation is an accepted method for the relief of fissure in ano, and in many cases gives very speedy results.

Dr. Walker recommends gradual dilatation for the following reasons:—

1. It is almost painless, at least after the first and second dilatation.

2. It does not tear the parts, nor does it produce paresis, as occasionally occurs after forcible dilatation.

3. Neither does it leave cicatrices, that are apt to produce strictures, as in the method of hypodermic injection or ligature of hæmorrhoids.—*The Medical Age*, August 25th, 1887.

**Salicylate of Soda in Gonorrhœal Orchitis.**—In the *Revue Mèdicale* for June, 1887, salicylate of soda is extolled as nearly a specific in gonorrhœal orchitis. At the end of some hours there will be a diminution of the pain, which disappears completely a short time afterwards. But it is in the acute cases of epididymitis, accompanied inflammation of the tunica vaginalis, that the treatment especially efficacious. It is without effect if the prominent symptom is inflammation of the cord. In the cases treated by the salicylate, the resolution of the swelling is more rapid, and can be obtained in from eight to ten days; and after a day or two the patient can get up. The remedy is recommended for its simplicity and harmlessness.—*Medical and Surgical Reporter*, September 24th, 1887. W. J. PENNY.

### LARYNGOLOGY.

**Cancer of the Larynx.**—Dr. H. A. Johnson publishes the details of five cases of this disease.

Case I. A male, aged 65, of good habits and no specific taint. The trouble in the throat began one year before consultation. Soon afterwards he had pain on swallowing, and the secretion of saliva became excessive and annoying. Dyspnœa and dysphonia supervened, and finally complete aphonia. A few attempts to reduce the tumour and relieve the dyspnœa by topical measures were made, but failed. One night, urgent dyspnœa compelled the performance of tracheotomy. The patient lived three months after the operation. The breathing was completely relieved, but deglutition remained painful. The tumour in this case was probably an epithelioma.

Case II. A male, aged 70; antecedents good; no cancer in the family. He suffered from dysphagia and marked dyspnœa. The vestibule of the larynx was filled with a ragged, irregular mass, with only a narrow, tortuous opening, through which a little air could pass. The patient declined operation, and died a few days afterwards during a paroxysm of dyspnœa.

Case III. Male, aged 68; antecedents good. He enjoyed good health until a year before consultation, when he began to have some irritability of the throat, and slight difficulty of swallowing, with cough. Examination revealed a somewhat smooth but slightly lobulated mass springing from the right internal wall above the vocal cords. Attempts were made to destroy the growth by galvano-cautery. Large masses were in this way destroyed, but growth was rapid. One month after the commencement of treatment the difficulty in breathing became worse; spasm of the respiratory muscles occurred; food and drink passed into the larynx, and on several occasions there appeared to be immediate danger to life. Tracheotomy was therefore performed, with relief of all the alarming symptoms. The patient lived eight months and a few days after the operation. The autopsy revealed an extensive epithelioma, involving the whole of the right half of the larynx, and also the left portion of the supra-glottic structures.

Case IV. Male, aged 48. In this case the swelling commenced, two years before consultation, outside the larynx. It gradually increased, and within the last few months the patient has had difficulty in speaking, and dyspnœa, especially at night. Examination with the laryngoscope revealed a smooth reddish tumefaction of the arytenoid, together with the supra-glottic structures on the same side. Tracheotomy was advised, but declined. The subsequent history of this case is not known.

Case V. Male, aged 54. His mother died of cancer, but antecedents otherwise good. General health good until one year before consultation, when he began to have some discomfort in the throat. For the few months before consultation this had been getting worse, with dysphagia and constant dyspnœa. Inspiration was more difficult than expiration, especially at night. There had been one severe hæmorrhage from the throat a few days before he was first seen by Dr. Johnson. Examination revealed a dark, reddish mass springing from the right side above the glottis, which bled on the slightest touch. Tracheotomy was performed, with marked relief to the breathing. The question of partial extirpation of the larynx was raised. The patient passed from observation for three months; when he returned, the growth had considerably increased in size, and the lymphatics on the right side of the neck were involved; and there had been frequent hæmorrhages. Under these circumstances, excision of the larynx was not performed, and the patient died soon after; *i.e.*, about five months after the tracheotomy.

In conclusion, Dr. Johnson calls attention to the following facts :—

1. That all the patients were males, the youngest 45 years old.

2. That of the five cases, the growth was evidently from the right side in three cases, from the left side in one case, and in the remaining case probably from the left side.

3. That in only one case was there a history of cancer in the family.

4. That in one case the disease appeared to be secondary, or an extension of an external cancer.

5. That in one case only was there any evidence of infection of the lymphatics from the larynx.

6. That in only one case was there any troublesome hæmorrhage.

7. That in none was there marked pain in the larynx.

8. That in three cases tracheotomy was performed, and the patients' lives were prolonged apparently three, five, and eight months respectively.—*New York Medical Journal*, September 24th, 1887.

**Unilateral Excision of the Larynx for Primary Carcinoma of the Larynx.**—Professor Socin removed half the larynx from a female patient, aged 50, for primary carcinoma. At the time of the operation the larynx was firmly adherent to the thyroid, and it was supposed that the disease had extended to that gland. After the larynx extirpation, the thyroid continued to increase rapidly, and was subsequently removed. She recovered well also from this operation, but has been unable to take food by the mouth, as, during deglutition, it passes into the larynx and causes asphyxia. She is fed exclusively by the stomach tube, and breathes through a tracheal tube. There has been great difficulty in keeping the larynx patent, as its lumen has become partly contracted by cicatricial tissue. Dilatation is now practised by passing bougies from the tracheal wound upwards.—*The Journal of the American Medical Association*, September 10th, 1887. W. J. PENNY.

